# Can we clinically diagnose dementia with Lewy bodies yet?

**DOI:** 10.1186/2047-9158-2-4

**Published:** 2013-02-11

**Authors:** Yue Huang, Glenda Halliday

**Affiliations:** 1 Neuroscience Research Australia, The University of New South Wales, Sydney, NSW, 2031, Australia

**Keywords:** Dementia with Lewy bodies, Diagnosis, Genetics, Pathogenesis, Pathology

## Abstract

Dementia with Lewy Bodies (DLB) was initially identified and confirmed primarily by pathology, but is soon to be incorporated into the Diagnostic and Statistical Manual criteria as a clinical disease entity. Despite these advances over more than 20 years, current data suggest that the sensitivity of accurate clinical diagnosis of DLB is still very low, although there is mounting evidence that supportive features may increase diagnostic accuracy. Although DLB remains easy to identify pathologically with different cellular pathologies differentiating it from other dementia syndromes, pathological identification using only Lewy body pathology has been shown to be inaccurate due to overlap with patients without dementia symptoms. A number of studies now suggest that a combination of cellular pathologies, which include α-synuclein and β-amyloid deposition as well as dopamine denervation, assist with differentiating this dementia syndrome from others. The clinical and pathological overlap with the tauopathy of Alzheimer’s disease still remains to be clarified. To determine more robust and independent clinicopathological correlates from Alzheimer’s disease, longitudinal prospective studies, using specific clinical batteries on dementia patients reaching the proposed criteria for DLB, with post-mortem assessment of the multiple pathologies associated with dementia, are required. Identifying genetic causes for DLB is another approach to investigate the pathogenesis of DLB. However this approach has been hindered to date by difficulties with identifying DLB clinically. The use of novel techniques is likely to advance knowledge on the pathogenesis of DLB and assist with redefining clinical and pathologic diagnostic criteria. To achieve the goal of more accurate clinical diagnosis of DLB, breakthroughs are necessary on the pathogenesis of DLB.

## Introduction

The Lewy body was named after Frederick Lewy who first described the abnormal intracytoplasm inclusions in 1912 in a patient with what is now called Parkinson’s disease (PD). In 1961, Okazaki first described a relationship between the presence of cortical Lewy bodies and dementia [1]. In 1984, Kosaka, Yoshimura, Ikeda and Budka reviewed the existing literature and proposed that these cases should be regarded as a new disease entity, which they termed ‘diffuse Lewy body disease’ [2]. Soon after the disease was confirmed by others [3,4]. The terminology of dementia with Lewy bodies (DLB) was proposed by scientists attending the 1996 proceedings of the First International Workshop of the Consortium on DLB [5]. Before 1996 when the term DLB was designated, there were several terms to describe the appearance of Lewy bodies in dementia, such as diffuse Lewy body disease, Lewy-body dementia, senile dementia of the Lewy body type, and dementia associated with cortical Lewy bodies [6]. As DLB cases have been included in other dementia subtypes (particularly Alzheimer’s disease or AD) in the past, its importance can only be assessed if the diagnostic criteria allow clear separation of the disorder from these other types of dementia. DLB is now considered part of a spectrum of Lewy body diseases, ie. PD, PD with dementia (PDD) and DLB [7,8], and is known to often coexist with AD (could be considered as a mixed dementia). To emphasize its clinical features, Japanese scientists proposed that DLB be called “Kosaka’s disease” [9], but this term is yet to be internationally accepted. In fact, DLB is not currently recognized as a diagnosis for dementia in the Diagnostic and Statistical Manual (DSM) for Mental Disorders published by the American Psychiatric Association, although there are plans for its incorporation into the 5^th^ version to be published in 2013.

The clinical criteria for possible and probable DLB diagnosis were last revised in 2005 following the 3^rd^ DLB International Workshop (Table 1) [8]. While the central feature of progressive cognitive decline that interferes with daily functioning is essential for diagnosis, the temporal sequence for diagnosis allows this feature to occur with progression in the context of a parkinsonian syndrome where it may only become prominent or persistent after the first few years. An arbitrary ‘one-year rule’ introduced at the same time as the disease entity in 1996 has been continued to be recommended to be applied to distinguish DLB from PDD: If dementia occurs within 12 months of the motor features, the patient should be classified as having a primary diagnosis of DLB [8], although the ‘one-year rule’ is not without challenge [7]. Additional core features required for diagnosis include fluctuating cognition with pronounced variations in attention and alertness, recurrent visual hallucinations that are well formed and detailed, and/or spontaneous features of parkinsonism. Possible DLB is identified if one of these features is present, while probable DLB is identified if two of these features are present. Three suggestive features were included in 2005 to assist with a diagnosis of DLB; rapid eye movement (REM) sleep behavior disorder, severe narcoleptic sensitivity and low dopamine transporter uptake in basal ganglia demonstrated by SPECT or PET. If one or more suggestive feature is present in addition to one core feature, then probable DLB can be diagnosed. If one or more suggestive feature is present in the absence of any core feature, then possible DLB can be diagnosed. The diagnosis of DLB is less likely 1) in the presence of cerebrovascular disease evident as focal neurologic signs or on brain imaging, 2) in the presence of any physical illness or brain disorder sufficient to account in part or in total for the clinical picture, and 3) if parkinsonism only appears for the first time at a severe stage of dementia. This last criterion appears to be the cause of much over-diagnosis of DLB in current clinical practice (see below).

**Table 1:** Revised criteria for the clinical diagnosis of DLB (2005)

***1. Central feature (essential for a diagnosis of possible or probable DLB)***	
	Dementia defined as progressive cognitive decline of sufficient magnitude to interfere with normal social or occupational function.
***2. Core features (two core features are sufficient for a diagnosis of probable DLB, and one for possible DLB)***	
	Fluctuating cognition with pronounced variations in attention and alertness
	Recurrent visual hallucinations that are typically well formed and detailed
	Spontaneous features of parkinsonism
***3. Suggestive features***	
	REM sleep behaviour disorder
	Severe neuroleptic sensitivity
	Low dopamine transporter uptake in basal ganglia demonstrated by SPECT or PET imaging.
***4. Supportive features***	
	Repeated falls and syncope
	Transient, unexplained loss of consciousness
	Severe autonomic dysfunction, e.g., orthostatic hypotension, urinary incontinence
	Hallucinations in other modalities
	Systematised delusions
	Depression
	Relative preservation of medial temporal lobe structures on CT/MRI scan
	Generalised low uptake on SPECT/PET perfusion scan with reduced occipital activity
	Abnormal (low uptake) MIBG myocardial scintigraphy
	Prominent slow wave activity on EEG with temporal lobe transient sharp waves
***5. A diagnosis of DLB is less likely***	
	In the presence of cerebrovascular disease evident as focal neurologic signs or on brain imaging
	In the presence of any other physical illness or brain disorder sufficient to account in part or in total for the clinical picture
	If parkinsonism only appears for the first time at a stage of severe dementia
***6. Temporal sequence of symptoms***	
	DLB should be diagnosed when dementia occurs concurrently or within one year of parkinsonism (if it is present).

The accuracy of the diagnostic criteria for DLB is variable depending on the populations assessed, with relatively low clinical sensitivity (lowest 12%, although 88% in recent work) but higher specificity (79-100%) [10-16]. There appears to be an increase in sensitivity with the inclusion of new supportive features discussed above [13,16]. In the six studies assessing the accuracy of clinical diagnoses since the 2005 criteria, one report selected cases where all clinical symptoms were assessed but determined the accuracy of diagnosis only in those 53 cases with a clinical diagnosis of DLB [12]. A second study of DLB patients assessed whether neuroimaging was of assistance in 20 cases who came to autopsy, finding that dopamine ligand imaging improved diagnosis significantly (88% sensitivity, 100% specificity in this small sample) [13]. A third study also assessed patients with Lewy bodies only and determined which pathology correlated most with the clinical phenotype, asserting that the severity of neurofibrillary tangles relate more to the diagnostic DLB criteria than Lewy bodies [10], consistent with previous suggestions [11]. This was confirmed in a fourth study assessing 304 people 85 years old and above [14]. A much larger series did not use the new supportive clinical features, but assessed clinical versus pathological diagnoses in 2,861 cases in the National Alzheimer’s Coordinating Center registry [15]. The accuracy of diagnosing clinical AD was higher (85% sensitivity, 51% specificity) than that for clinical DLB (32% sensitivity for pure and 12% for mixed, 95% specificity). However, the most recent study assessing 234 consecutive patients with dementia coming to autopsy has shown that including REM sleep behavior disorder significantly improves the diagnostic accuracy of clinical DLB [16]. The still significant clinical under-detection of probable DLB is discussed in this review. In addition, we review the neuropathology of DLB and its overlapping pathology with other disorders, as well as the genetics and pathogenesis of DLB. We expect that as cellular and molecular knowledge on DLB advances, the diagnostic criteria for DLB will undergo further modifications.

## Clinical presentation and differentiation of DLB

As stated above, significant cognitive decline is mandatory for the diagnosis of DLB. Patients with DLB usually have deficits on tests of attention, executive function (e.g. planning, prioritizing, and sequencing) and visuospatial ability. In a cross-sectional clinical study [17], memory impairment (57%) was the most common presenting symptom in DLB, followed by visual hallucinations (44%), depression (34%), problem solving difficulties (33%), gait problems (28%), and tremor/stiffness (25%). In contrast, 99% of AD carers reported impaired memory as a presenting symptom, whereas visual hallucinations were a presenting symptom in 3% of the AD cases [17]. These observations support previous suggestions that DLB should be suspected in mild memory impairment cases with visual hallucinations [18], although this feature is not highly prevalent in patients with autopsy confirmed DLB [17]. In community studies [6,19], DLB accounts for approximately 0.7% of the general population older than 65 years of age, and 5% (3.3% probable, 1.7% possible) of people over 85 years of age. Hospital-based autopsy series report 10-15% of patients with DLB and population-based autopsy series show Lewy body pathology evenly distributed between demented and non-demented individuals, suggesting a substantial pool of preclinical cases [6]. This has been confirmed in more recent, expansive autopsy series [20]. The early identification of DLB, as well as the differentiation of DLB from other conditions, has become very important to determine treatment options, help with behavioral management of patients, and provide informative resources for caregivers.

### Fluctuating cognition

Fluctuations may reflect periods of unresponsiveness while awake (e.g., blanking out, zoning out), episodes of excessive somnolence despite adequate night-time sleep, or periods of daytime behavioural confusion with limited awareness of surroundings alternating with normal or near normal function. Cognitive fluctuations have also been described as variability in the patients’ cognitive or functional abilities and periods when the patient regains their ability to perform tasks that they were previously unable to perform. However, studies show that fluctuations in cognition are problematic to define and it is hard to obtain consistent assessments for their presence [11,21], which has been proposed as the main factor for low sensitivity of the current clinical diagnosis criteria [22]. To overcome this difficulty, there are several clinical tests available for the evaluation of fluctuations: Clinician Assessment of Fluctuation scale, semi-structured One Day Fluctuation Assessment scale, and The Mayo Fluctuations Composite Scale [11,23]. However, these tools have yet to be evaluated for their reliability and validity [24]. As the prevalence of this feature is high in autopsy confirmed cases of DLB, clear and reliable assessment of fluctuating cognition is warranted.

### Recurrent visual hallucination

In DLB, visual hallucinations are typically recurrent and well formed, consisting of three dimensional subjects, people, children or animals. Visual misperceptions (illusions) are also common [6,19,25]. Examples of object misperceptions in DLB include mistaking a hinge for a caterpillar, or patterns on a rug for snakes, etc. Auditory hallucinations and delusional ideation can occur in a subgroup of those with visual hallucinations [26]. The most frequent delusional ideas include strangers or intruders in the home or the belief that deceased friends or family members are visiting, and examples of elementary auditory hallucinations include banging, knocking, sizzling, a doorbell or footsteps. These are associated with Lewy body counts in the anterior and inferior temporal lobe, claustrum and the amygdala, regions previously associated with complex visual image generation [27-30]. The presence of visual hallucinations and global cognitive impairment are also associated with deficits in choline acetyltransferase (ChAT) in DLB. ChAT is an indicator of neocortical cholinergic activity, which is more depleted in DLB than in AD [31]. It is unknown whether lower levels of ChAT correlate with greater Lewy body loads in DLB. The Neuropsychiatric Inventory (NPI) can be used in the clinic to screen visual hallucinations and assess their severity and frequency [32].

### Spontaneous parkinsonism

The extrapyramidal signs should be spontaneous or occur subsequent to dementia in DLB [7], although dementia is allowed to occur within one year of earlier parkinsonism according to the current temporal designation of DLB [8], as discussed above. Postural instability, gait difficulty, and facial immobility are axial features common in DLB, while rest tremor of the limbs is less common. Levodopa responsiveness in DLB is also less common [33,34]. The severity of motor features in DLB can be assessed using the Unified Parkinson's Disease Rating Scale (UPDRS) [8].

### Rapid eye movement (REM) sleep behavior disorder

REM sleep behavior disorder can develop in otherwise neurologically-normal adults as well as in those with a neurodegenerative disease [35]. REM sleep behavior disorder has been included as a supportive clinical feature for DLB and has been shown to enhance the diagnosis of DLB [16], possibly because it is a significant risk factor for both cognitive deficits and PD [36]. DLB patients with probable REM sleep behavior disorder have an earlier onset of other symptoms (parkinsonism and visual hallucinations), a more rapid disease course and less AD but similar Lewy-related pathologies compared with DLB patients who do not have REM sleep behaviour disorder [37]. These observations suggest that REM sleep behavior disorder in patients with DLB may indicate a particular subtype of DLB.

### Severe neuroleptic sensitivity

About 30 to 50% of DLB patients who take antipsychotic drugs (olanzapine – 58%, clozapine – 11%, thioridazine – 6%) will develop severe neuroleptic sensitivity, manifested as a sudden onset of drowsiness, increased confusion, immobility, and muscle rigidity [38,39]. Severe neuroleptic sensitivity is not seen in patients with AD, but occurs in 27% of patients with PD, 39% with PDD, and is most frequent in patients with DLB (53%) [38]. It has been proposed that this change in sensitivity is due to dysfunction of dopamine D2 receptors [38]. As severe neuroleptic sensitivity reactions can be fatal, more research and education on this issue needs to be conducted [39].

### Low dopamine transporter uptake in basal ganglia demonstrated by SPECT or PET

Low dopamine transporter uptake in the basal ganglia, measured with (123)I-FP-CIT (DaTSCAN) SPECT imaging or (18) fluorodopa PET, is clinically useful in distinguishing DLB from AD [40,41]. A retrospective study showed that DaTSCAN findings were concordant with the outcome clinical DLB diagnosis in 95% cases [42]. A systematic meta-analysis of published studies on DLB diagnostic accuracy of presynaptic dopaminergic imaging with DaTSCAN revealed 86.5% sensitivity and 93.6% specificity differentiating DLB from non-DLB [43].

### Other imaging characteristics

Other supportive features include abnormal cardiac sympathetic imaging using Iodine-123 Metaiodobenzilguanidine (I-123 MIBG), occipital and posterior parietotemporal lobe hypometabolism [44], and relative preservation of medial temporal lobe structures [45], etc. (Table 1).

### Differentiation

AD and PDD are the two major disorders required to be clinically differentiated from DLB. The most difficult syndrome to differentiate from DLB is AD, as prominent or persistent memory impairment occurs in the early stages of both disorders. In addition, DLB patients with concomitant AD are less likely to have hallucinations compared to patients with pure DLB, possibly because the clinical DLB features are masked by the clinical features of AD [11]. A further difficulty has been that the characteristic features of DLB can often manifest in AD patients when the dementia has progressed to a severe stage, and thus the majority of patients diagnosed with DLB have had severe AD rather than DLB or mixed DLB and AD pathologically [15]. These data show that the core clinical features of DLB are only effective for clinical differentiation from AD at the onset of disease [46]. This problem with the overlap with pathologically proven AD may be assisted with the inclusion of some of the supportive features for DLB [16], but this needs to be evaluated further. A recent study suggests that the assessment of non-motor symptoms associated with PD (olfactory dysfunction, constipation, increased saliva and signs of rapid eye movement sleep behavior disorder) at the onset of dementia can assist with differentiating DLB from AD [47]. Such non-motor symptoms are being actively studied in large clinical cohorts as prodromes for PD [48] with research on potential prodromal features for DLB also currently underway [49]. Such research may provide important yet-to-be-defined differentiating features.

Differentiating PDD from DLB is currently an arbitrary construct, but at least has proven less problematic. PDD in the context of well-established PD when subsequent dementia becomes evident is easily differentiable from the dominant dementia syndrome observed in most cases of DLB. The clinical diagnostic criteria for PDD have been published and operationalised and use the three core features of DLB [50,51]. While the clinical diagnostic criteria for "probable" and "possible" PDD has been proposed with a simple, pragmatic set of tests involved [50], the sensitivity (47%) of these tests for diagnosing dementia in the context of PD is low [52]. While the differential diagnosis between DLB and PDD is not problematic due to the artificial construct, the identification of all dementia cases in patients with PD needs to be improved.

## Pathological manifestations and differentiation of DLB

**Figure 1:**
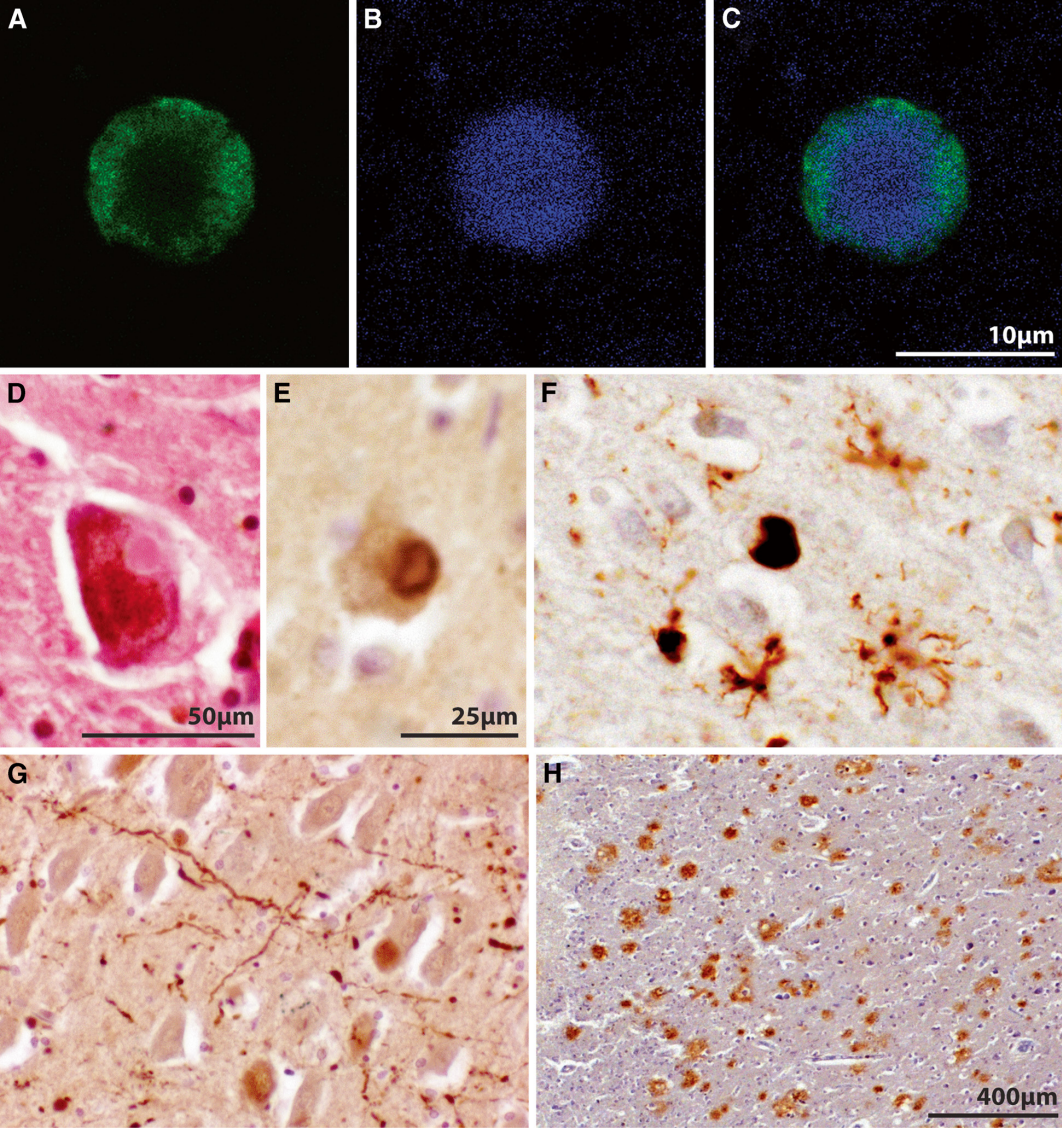
**Lewy-related pathologies (LRP) and Aβ deposition in DLB.** (A-C) Histofluorecence identification of α-synuclein-positive (**A**, green) Lewy bodies using thioflavin S (**B**, blue) for filament localization (**C**, merge) showing difficulty with immunostaining the mature core filaments of midbrain Lewy bodies. (**D**) Eosinophilic Lewy body in a pigmented midbrain dopaminergic neuron, (**E**) mature α-synuclein-positive Lewy body in the neocortex identified using peroxidase immunohistochemistry, (**F**) immature α-synuclein-positive Lewy body and related star-shaped α-synuclein-positive astrocytes in the neocortex identified using peroxidase immunohistochemistry. (**G**) α-synuclein-positive Lewy neurites in the hippocampal CA2 region identified using peroxidase immunohistochemistry, and (**H**) Aβ-positive cortical plaques identified using peroxidase immunohistochemistry. The scale in F and G is equal to that in E.

The diagnosis of DLB is based on assessing the probability that the pattern and degree of Lewy-related pathology (LRP) versus AD pathology is related to a cognitive disorder [19,53]. Currently, pure DLB is diagnosed pathologically according to the severity and distribution of LRP in certain brain regions, and Braak neurofibrillary stages less than stage IV [54,55]. Fibrillar forms of α-synuclein are the major component of LRP (Figure 1) [56]. At end stage, α-synuclein LRP is diffusely distributed in cortical and subcortical regions in both DLB and PDD [6,57]. α-Synuclein-immunoreactive astrocytes are also noted in PDD as well as in DLB (Figure 1) [58]. However, novel techniques including paraffin-embedded tissue blotting and protein aggregate filtration assays reveal substantial accumulation of α-synuclein aggregates in presynapses in DLB [59], suggesting that presynaptic α-synuclein aggregates, rather LRP, cause neurodegeneration. Whether such techniques would assist with differential diagnosis needs further examination.

At present there is a lot of controversy over the clinical significance of LRP and so further refinement of techniques in this area may be warranted. Patients with pathologically-confirmed DLB are not all demented; 40-80% of those have visual hallucinations, 50-80% develop parkinsonism, and overall about 30-50% of subjects demonstrate both visual hallucination and parkinsonism in retrospective studies [11,50]. In one pathological sample, patients satisfying the McKeith criteria for diffuse neocortical DLB with only mild concomitant AD pathology were demented only 48% of the time with only 54% displaying extrapyramidal symptoms [20]. However, these clinical findings were gathered retrospectively questioning whether the clinical assessment of these patients was sufficiently detailed to discount the presence of other DLB features prior to autopsy. On the other hand, widespread LRP occurs in many sporadic AD cases without parkinsonian symptoms [60], sometimes reaching pathological criteria for both DLB and AD [61]. In particular, familial DLB cases often show concomitant DLB and AD [61]. Retrospective studies show that DLB + AD patients have longer disease durations compared to patients with pure DLB pathology [11]. Further population-based, longitudinal prospective clinical and pathological correlation studies are warranted to determine the clinical significance of LRP in both the normal and dementia populations.

In comparison to LRP, β-amyloid plaques are often seen in DLB compared to PDD [62-65]. Unlike AD, β-amyloid plaques in pure DLB are diffuse and without tau neuritic involvement [17]. Whether or not β-amyloid deposition contributes to any DLB clinical feature particularly, or more to the timing and/or severity of cognitive decline, remains to be determined. The common occurrence of β-amyloid deposition in the setting of clinical DLB may prove more difficult in terms of differentiation from AD, although as discussed above, the assessment of imaging for dopamine transporters seems to assist with the differential diagnosis from AD [66]. This highlights another important pathological aspect to assist with case differentiation, the pattern of cell loss or atrophy. On this aspect, not only can imaging dopamine cell loss assist, but there is also more limited cortical and hippocampal atrophy in DLB versus AD [44]. There is even more limited cell death in PDD, with hippocampal sparing in comparison to DLB [28,67]. The overlap between these disorders can best be seen as a pathological continuum with dopamine cell loss and widespread LRP observed in both PDD and DLB, with additional β-amyloid deposition and mild cortical and hippocampal atrophy observed in pure DLB, and with greater hippocampal and cortical degeneration associated with additional tau deposition observed in mixed DLB with AD, while AD has the β-amyloid and tau deposition without the widespread LRP. This also highlights the necessity to assess more than one pathological feature in diagnosing these complex syndromes.

## Genetics of DLB

By analysing the incidence of familial and sporadic DLB cases in autopsy-confirmed series, a family history of dementia is more common in DLB compared with controls [68] and can run in families in an autosomal dominant inheritance pattern [69]. These studies indicate a genetic contribution to this disorder. Variants in all 3 members of the synuclein gene family, α-, β-, and γ-synuclein affect the risk of developing DLB [70].

γ-Synuclein, aberrantly expressed in many different malignant tumours, is primarily found in the peripheral nervous system and also detected in brain. β-Synuclein, the non-amyloidogenic homolog of α-synuclein, is associated with DLB and not PD. V70M and P123H mutations in the β-synuclein gene have been shown to predispose to DLB [71]. In contrast, genetic mutations in the α-synuclein gene are most commonly associated with PD, with or without dementia. However, an E46K mutation in the α-synuclein gene has been found in one Spanish family with DLB [72] and triplication of the α-synuclein gene occurs in a Swedish American family with early-onset parkinsonism and dementia [73], consistent with current DLB diagnostic criteria. However, mutations in the α-synuclein gene are very rare in familial patients with either PD or DLB [74,75]. Common genetic factors for the susceptibility of these LRP disorders have been identified in the glucocerebrosidase (GBA) gene [76]. A significantly higher heterozygote frequency for two common GBA gene mutations (N370S and L444P) occurs in patients with PD (2.9%; P <.001) or DLB (3.5%; P = .045) compared with control subjects (0.4%) [77].

A novel locus for DLB has been identified in Belgian families [78]. A genome-wide scan and subsequent fine mapping of candidate loci revealed the locus linkage to 2q35-q36 adjacent to the previously reported PARK11 locus. Screening five candidate genes has not identified the disease-causing mutation [78]. Further analysis of AD and PD causative genes in patients with DLB has been performed showing copy number variants in *APP*, *SNCA* and *PARK2* genes [76]. However, as the clinical diagnosis of DLB remains difficult, whether current genes associated with either AD or PD pathology influence all aspects of DLB or more particularly some overlapping pathology, remains to be determined. Due to poor sensitivity of the clinical diagnosis of DLB, identification of genes causing DLB requires autopsy-confirmation, rendering such studies difficult. Genetic analysis on further families with multiple autopsies is currently the only way for breakthroughs in the genetics of DLB.

## Conclusions

There is a need for better clinical differentiation of people who have a distinct pathological process from PD or AD – that is LRP with β-amyloid deposition but not overt AD tau pathology. There is also a need to determine the clinical consequences of LRP deposition alone without overt parkinsonism or dementia, and the risk factors for this prodrome. Better definitions and more sensitive diagnostic procedures are vital for accurate estimation of prevalence and incidence of DLB across different populations. Finally, identifying causative genes in a better defined population of DLB cases is likely to provide a promising way forward to shed light on any potential different pathogenic mechanism/s for DLB, and assist with increasing the accuracy of clinical diagnosis for this overlapping dementia syndrome.

## Abbreviations

(123)I-FP-CIT: (123)I-2beta-carbometoxy-3beta-(4-iodophenyl)-N-(3-fluoropropyl) nortropane; AD: Alzheimer’s disease; DLB: Dementia with Lewy Bodies; DSM: Diagnostic and Statistical Manual; I-123 MIBG: Iodine-123 Metaiodobenzilguanidine; LRP: Lewy-related pathology; PD: Parkinson’s disease; PDD: Parkinson’s disease with dementia.

## Competing interests

There are no financial competing interests to declare in relation to this manuscript.

## Authors’ contributions

YH drafted the manuscript. GMH conceived the structure of the manuscript & revised the manuscript. Both authors read and approved the final manuscript.

## Authors’ information

**YH** trained as a neurologist in China with nine years of clinical practice experience and ten years of medical research in Australia. YH currently works as a senior research officer at Neuroscience Research Australia & is a conjoint senior lecturer at the University of New South Wales, Australia.

**GMH** is an expert in the pathogenesis of Parkinson’s disease and other neurodegenerative disorders. She received her degrees from the University of New South Wales and postdoctoral training at the Centre for Neuroscience, Flinders University of South Australia prior to returning to Sydney as an Australian Research Council Queen Elizabeth II Fellow. She has been a research fellow of the National Health and Medical Research Council of Australia since then and one of the senior scientists at Neuroscience Research Australia (joined in 1993). She has published over 300 research articles and two books, the most recent on Parkinson’s disease, and was president of the Australian Neuroscience Society from 2006–2007. Her pathological work on dementia with Lewy bodies has been incorporated into highly cited research criteria for the diagnosis of this disorder, highlighting the association between Lewy body deposition and visual hallucinations rather than a loss of function.

## References

[B1] Okazaki H Lipkin LE Aronson SM Diffuse intracytoplasmic ganglionic inclusions (Lewy type) associated with progressive dementia and quadriparesis in flexion J Neuropathol Exp Neurol 1961 20 237 244 10.1097/00005072-196104000-00007 13730588

[B2] Kosaka K Yoshimura M Ikeda K Budka H Diffuse type of Lewy body disease: progressive dementia with abundant cortical Lewy bodies and senile changes of varying degree–a new disease? Clin Neuropathol 1984 3 185 192 6094067

[B3] Dickson DW Davies P Mayeux R Crystal H Horoupian DS Thompson A Goldman JE Diffuse Lewy body disease. Neuropathological and biochemical studies of six patients Acta Neuropathol 1987 75 8 15 10.1007/BF00686786 3434218

[B4] Hansen L Salmon D Galasko D Masliah E Katzman R DeTeresa R Thal L Pay MM Hofstetter R Klauber M The Lewy body variant of Alzheimer's disease: a clinical and pathologic entity Neurology 1990 40 1 8 2153271 10.1212/wnl.40.1.1

[B5] McKeith IG Galasko D Kosaka K Perry EK Dickson DW Hansen LA Salmon DP Lowe J Mirra SS Byrne EJ Consensus guidelines for the clinical and pathologic diagnosis of dementia with Lewy bodies (DLB): report of the consortium on DLB international workshop Neurology 1996 47 1113 1124 10.1212/WNL.47.5.1113 8909416

[B6] McKeith I Mintzer J Aarsland D Burn D Chiu H Cohen-Mansfield J Dickson D Dubois B Duda JE Feldman H Dementia with Lewy bodies Lancet Neurol 2004 3 19 28 10.1016/S1474-4422(03)00619-7 14693108

[B7] McKeith I Dementia with Lewy bodies and Parkinson's disease with dementia: where two worlds collide Pract Neurol 2007 7 374 382 10.1136/jnnp.2007.134163 18024777

[B8] McKeith IG Dickson DW Lowe J Emre M O'Brien JT Feldman H Cummings J Duda JE Lippa C Perry EK Diagnosis and management of dementia with Lewy bodies: third report of the DLB Consortium Neurology 2005 65 1863 1872 10.1212/01.wnl.0000187889.17253.b1 16237129

[B9] Kosaka K Kosaka's disease Shinkei kenkyu no shinpo 2008 60 13 16 18232328

[B10] Weisman D Cho M Taylor C Adame A Thal LJ Hansen LA In dementia with Lewy bodies, Braak stage determines phenotype, not Lewy body distribution Neurology 2007 69 356 359 10.1212/01.wnl.0000266626.64913.0f 17646627

[B11] Merdes AR Hansen LA Jeste DV Galasko D Hofstetter CR Ho GJ Thal LJ Corey-Bloom J Influence of Alzheimer pathology on clinical diagnostic accuracy in dementia with Lewy bodies Neurology 2003 60 1586 1590 10.1212/01.WNL.0000065889.42856.F2 12771246

[B12] Fujishiro H Ferman TJ Boeve BF Smith GE Graff-Radford NR Uitti RJ Wszolek ZK Knopman DS Petersen RC Parisi JE Dickson DW Validation of the neuropathologic criteria of the third consortium for dementia with Lewy bodies for prospectively diagnosed cases J Neuropathol Exp Neurol 2008 67 649 656 10.1097/NEN.0b013e31817d7a1d 18596548 PMC2745052

[B13] Walker Z Jaros E Walker RW Lee L Costa DC Livingston G Ince PG Perry R McKeith I Katona CL Dementia with Lewy bodies: a comparison of clinical diagnosis, FP-CIT single photon emission computed tomography imaging and autopsy J Neurol Neurosurg Psychiatry 2007 78 1176 1181 10.1136/jnnp.2006.110122 17353255 PMC2117602

[B14] Oinas M Polvikoski T Sulkava R Myllykangas L Juva K Notkola IL Rastas S Niinisto L Kalimo H Paetau A Neuropathologic findings of dementia with lewy bodies (DLB) in a population-based Vantaa 85+ study J Alzheim Dis 2009 18 677 689 10.3233/JAD-2009-1169 19625740

[B15] Nelson PT Jicha GA Kryscio RJ Abner EL Schmitt FA Cooper G Xu LO Smith CD Markesbery WR Low sensitivity in clinical diagnoses of dementia with Lewy bodies J Neurol 2010 257 359 366 10.1007/s00415-009-5324-y 19795154 PMC2839040

[B16] Ferman TJ Boeve BF Smith GE Lin SC Silber MH Pedraza O Wszolek Z Graff-Radford NR Uitti R Van Gerpen J Inclusion of RBD improves the diagnostic classification of dementia with Lewy bodies Neurology 2011 77 875 882 10.1212/WNL.0b013e31822c9148 21849645 PMC3162640

[B17] Auning E Rongve A Fladby T Booij J Hortobagyi T Siepel FJ Ballard C Aarsland D Early and presenting symptoms of dementia with lewy bodies Dement Geriatr Cogn Disord 2011 32 202 208 10.1159/000333072 22095040

[B18] Tiraboschi P Salmon DP Hansen LA Hofstetter RC Thal LJ Corey-Bloom J What best differentiates Lewy body from Alzheimer's disease in early-stage dementia? Brain 2006 129 729 735 10.1093/brain/awh725 16401618

[B19] McKeith IG Consensus guidelines for the clinical and pathologic diagnosis of dementia with Lewy bodies (DLB): report of the Consortium on DLB International Workshop J Alzheim Dis 2006 9 417 423 10.3233/jad-2006-9s347 16914880

[B20] Parkkinen L Pirttila T Alafuzoff I Applicability of current staging/categorization of alpha-synuclein pathology and their clinical relevance Acta Neuropathol 2008 115 399 407 10.1007/s00401-008-0346-6 18297293 PMC2270355

[B21] Verghese J Crystal HA Dickson DW Lipton RB Validity of clinical criteria for the diagnosis of dementia with Lewy bodies Neurology 1999 53 1974 1982 10.1212/WNL.53.9.1974 10599768

[B22] McKeith IG Dementia with Lewy bodies Br J Psychiatr 2002 180 144 147 10.1192/bjp.180.2.144 11823325

[B23] Ferman TJ Smith GE Boeve BF Ivnik RJ Petersen RC Knopman D Graff-Radford N Parisi J Dickson DW DLB fluctuations: specific features that reliably differentiate DLB from AD and normal aging Neurology 2004 62 181 187 10.1212/WNL.62.2.181 14745051

[B24] Lee DR Taylor JP Thomas AJ Assessment of cognitive fluctuation in dementia: a systematic review of the literature Int J Geriatr Psychiatry 2012 27 989 998 10.1002/gps.2823 22278997

[B25] Uchiyama M Nishio Y Yokoi K Hirayama K Imamura T Shimomura T Mori E Pareidolias: complex visual illusions in dementia with Lewy bodies Brain 2012 135 2458 2469 10.1093/brain/aws126 22649179 PMC3407420

[B26] Bonanni L Thomas A Tiraboschi P Perfetti B Varanese S Onofrj M EEG comparisons in early Alzheimer's disease, dementia with Lewy bodies and Parkinson's disease with dementia patients with a 2-year follow-up Brain 2008 131 690 705 10.1093/brain/awm322 18202105

[B27] Harding AJ Broe GA Halliday GM Visual hallucinations in Lewy body disease relate to Lewy bodies in the temporal lobe Brain 2002 125 391 403 10.1093/brain/awf033 11844739

[B28] Harding AJ Lakay B Halliday GM Selective hippocampal neuron loss in dementia with Lewy bodies Ann Neurol 2002 51 125 128 10.1002/ana.10071 11782993

[B29] Yamamoto R Iseki E Murayama N Minegishi M Marui W Togo T Katsuse O Kosaka K Kato M Iwatsubo T Arai H Correlation in Lewy pathology between the claustrum and visual areas in brains of dementia with Lewy bodies Neurosci Lett 2007 415 219 224 10.1016/j.neulet.2007.01.029 17275187

[B30] Yamamoto R Iseki E Murayama N Minegishi M Marui W Togo T Katsuse O Kato M Iwatsubo T Kosaka K Arai H Investigation of Lewy pathology in the visual pathway of brains of dementia with Lewy bodies J Neurol Sci 2006 246 95 101 10.1016/j.jns.2006.02.016 16624323

[B31] Tiraboschi P Hansen LA Alford M Sabbagh MN Schoos B Masliah E Thal LJ Corey-Bloom J Cholinergic dysfunction in diseases with Lewy bodies Neurology 2000 54 407 411 10.1212/WNL.54.2.407 10668703

[B32] Cummings JL Mega M Gray K Rosenberg-Thompson S Carusi DA Gornbein J The Neuropsychiatric Inventory: comprehensive assessment of psychopathology in dementia Neurology 1994 44 2308 2314 10.1212/WNL.44.12.2308 7991117

[B33] Molloy S McKeith IG O'Brien JT Burn DJ The role of levodopa in the management of dementia with Lewy bodies J Neurol Neurosurg Psychiatry 2005 76 1200 1203 10.1136/jnnp.2004.052332 16107351 PMC1739807

[B34] Lucetti C Logi C Del Dotto P Berti C Ceravolo R Baldacci F Dolciotti C Gambaccini G Rossi G Bonuccelli U Levodopa response in dementia with lewy bodies: a 1-year follow-up study Parkinsonism Relat Disord 2010 16 522 526 10.1016/j.parkreldis.2010.06.004 20615745

[B35] Boeve BF Ferman TJ Neuropsychological characterization of evolving cognitive decline in idiopathic REM sleep behavior disorder is important, but not easy Sleep 2011 34 561 562 21532947 10.1093/sleep/34.5.561 PMC3079933

[B36] Boot BP Boeve BF Roberts RO Ferman TJ Geda YE Pankratz VS Ivnik RJ Smith GE McDade E Christianson TJ Probable rapid eye movement sleep behavior disorder increases risk for mild cognitive impairment and Parkinson disease: a population-based study Ann Neurol 2012 71 49 56 10.1002/ana.22655 22275251 PMC3270692

[B37] Dugger BN Boeve BF Murray ME Parisi JE Fujishiro H Dickson DW Ferman TJ Rapid eye movement sleep behavior disorder and subtypes in autopsy-confirmed dementia with Lewy bodies Mov Disord 2012 27 72 78 10.1002/mds.24003 22038951 PMC3513369

[B38] Aarsland D Perry R Larsen JP McKeith IG O'Brien JT Perry EK Burn D Ballard CG Neuroleptic sensitivity in Parkinson's disease and parkinsonian dementias J Clin Psychiatry 2005 66 633 637 15889951

[B39] Hassan I Consultation-liaison psychiatry and prevention of severe neuroleptic sensitivity reactions in dementia with Lewy bodies Australas Psychiatr 2011 19 536 537 10.3109/10398562.2011.580750 22077306

[B40] McKeith I O'Brien J Walker Z Tatsch K Booij J Darcourt J Padovani A Giubbini R Bonuccelli U Volterrani D Sensitivity and specificity of dopamine transporter imaging with 123I-FP-CIT SPECT in dementia with Lewy bodies: a phase III, multicentre study Lancet Neurol 2007 6 305 313 10.1016/S1474-4422(07)70057-1 17362834

[B41] Klein JC Eggers C Kalbe E Weisenbach S Hohmann C Vollmar S Baudrexel S Diederich NJ Heiss WD Hilker R Neurotransmitter changes in dementia with Lewy bodies and Parkinson disease dementia in vivo Neurology 2010 74 885 892 10.1212/WNL.0b013e3181d55f61 20181924

[B42] Kemp PM Clyde K Holmes C Impact of 123I-FP-CIT (DaTSCAN) SPECT on the diagnosis and management of patients with dementia with Lewy bodies: a retrospective study Nucl Med Commun 2011 32 298 302 10.1097/MNM.0b013e328343d4ec 21278615

[B43] Papathanasiou ND Boutsiadis A Dickson J Bomanji JB Diagnostic accuracy of (1)(2)(3)I-FP-CIT (DaTSCAN) in dementia with Lewy bodies: a meta-analysis of published studies Parkinsonism Relat Disord 2012 18 225 229 10.1016/j.parkreldis.2011.09.015 21975260

[B44] Kantarci K Lowe VJ Boeve BF Weigand SD Senjem ML Przybelski SA Dickson DW Parisi JE Knopman DS Smith GE Multimodality imaging characteristics of dementia with Lewy bodies Neurobiol Aging 2012 33 2091 2105 10.1016/j.neurobiolaging.2011.09.024 22018896 PMC3288845

[B45] Vemuri P Simon G Kantarci K Whitwell JL Senjem ML Przybelski SA Gunter JL Josephs KA Knopman DS Boeve BF Antemortem differential diagnosis of dementia pathology using structural MRI: Differential-STAND Neuroimage 2011 55 522 531 10.1016/j.neuroimage.2010.12.073 21195775 PMC3039279

[B46] Boeve BF Molano JR Ferman TJ Smith GE Lin SC Bieniek K Haidar W Tippmann-Peikert M Knopman DS Graff-Radford NR Validation of the Mayo Sleep Questionnaire to screen for REM sleep behavior disorder in an aging and dementia cohort Sleep Med 2011 12 445 453 10.1016/j.sleep.2010.12.009 21349763 PMC3083495

[B47] Chiba Y Fujishiro H Iseki E Ota K Kasanuki K Hirayasu Y Satoa K Retrospective survey of prodromal symptoms in dementia with Lewy bodies: comparison with Alzheimer's disease Dement Geriatr Cogn Disord 2012 33 273 281 10.1159/000339363 22722638

[B48] Olanow CW Obeso JA The significance of defining preclinical or prodromal Parkinson's disease Mov Disord 2012 27 666 669 10.1002/mds.25019 22508285

[B49] Jicha GA Schmitt FA Abner E Nelson PT Cooper GE Smith CD Markesbery WR Prodromal clinical manifestations of neuropathologically confirmed Lewy body disease Neurobiol Aging 2010 31 1805 1813 10.1016/j.neurobiolaging.2008.09.017 19026468 PMC2891418

[B50] Dubois B Burn D Goetz C Aarsland D Brown RG Broe GA Dickson D Duyckaerts C Cummings J Gauthier S Diagnostic procedures for Parkinson's disease dementia: recommendations from the movement disorder society task force Mov Disord 2007 22 2314 2324 10.1002/mds.21844 18098298

[B51] Emre M Aarsland D Brown R Burn DJ Duyckaerts C Mizuno Y Broe GA Cummings J Dickson DW Gauthier S Clinical diagnostic criteria for dementia associated with Parkinson's disease Mov Disord 2007 22 1689 1707 quiz 1837 10.1002/mds.21507 17542011

[B52] Barton B Grabli D Bernard B Czernecki V Goldman JG Stebbins G Dubois B Goetz CG Clinical validation of Movement Disorder Society-recommended diagnostic criteria for Parkinson's disease with dementia Mov Disord 2012 27 248 253 10.1002/mds.24059 22162144

[B53] Dickson DW Braak H Duda JE Duyckaerts C Gasser T Halliday GM Hardy J Leverenz JB Del Tredici K Wszolek ZK Litvan I Neuropathological assessment of Parkinson's disease: refining the diagnostic criteria Lancet Neurol 2009 8 1150 1157 10.1016/S1474-4422(09)70238-8 19909913

[B54] McKeith IG Rowan E Askew K Naidu A Allan L Barnett N Lett D Mosimann UP Burn D O'Brien JT More severe functional impairment in dementia with lewy bodies than Alzheimer disease is related to extrapyramidal motor dysfunction Am J Geriatr Psychiatr 2006 14 582 588 10.1097/01.JGP.0000216177.08010.f4 16816011

[B55] Dickson DW Neuropathology of non-Alzheimer degenerative disorders Int J Clin Exp Pathol 2009 3 1 23 19918325 PMC2776269

[B56] Wakabayashi K Matsumoto K Takayama K Yoshimoto M Takahashi H NACP, a presynaptic protein, immunoreactivity in Lewy bodies in Parkinson's disease Neurosci Lett 1997 239 45 48 10.1016/S0304-3940(97)00891-4 9547168

[B57] Braak H Ghebremedhin E Rub U Bratzke H Del Tredici K Stages in the development of Parkinson's disease-related pathology Cell Tissue Res 2004 318 121 134 10.1007/s00441-004-0956-9 15338272

[B58] Braak H Sastre M Del Tredici K Development of alpha-synuclein immunoreactive astrocytes in the forebrain parallels stages of intraneuronal pathology in sporadic Parkinson's disease Acta Neuropathol 2007 114 231 241 10.1007/s00401-007-0244-3 17576580

[B59] Kramer ML Schulz-Schaeffer WJ Presynaptic alpha-synuclein aggregates, not Lewy bodies, cause neurodegeneration in dementia with Lewy bodies J Neurosci 2007 27 1405 1410 10.1523/JNEUROSCI.4564-06.2007 17287515 PMC6673583

[B60] Jellinger KA Alpha-synuclein pathology in Parkinson's and Alzheimer's disease brain: incidence and topographic distribution–a pilot study Acta Neuropathol 2003 106 191 201 10.1007/s00401-003-0725-y 12845452

[B61] Walker Z McKeith I Rodda J Qassem T Tatsch K Booij J Darcourt J O'Brien J Comparison of cognitive decline between dementia with Lewy bodies and Alzheimer's disease: a cohort study BMJ Open 2012 2 e000380 10.1136/bmjopen-2011-000380 PMC3330257 22318660

[B62] Halliday GM Song YJ Harding AJ Striatal beta-amyloid in dementia with Lewy bodies but not Parkinson's disease J Neural Transm 2011 118 713 719 10.1007/s00702-011-0641-6 21479514

[B63] Gomperts SN Rentz DM Moran E Becker JA Locascio JJ Klunk WE Mathis CA Elmaleh DR Shoup T Fischman AJ Imaging amyloid deposition in Lewy body diseases Neurology 2008 71 903 910 10.1212/01.wnl.0000326146.60732.d6 18794492 PMC2637553

[B64] Kalaitzakis ME Walls AJ Pearce RK Gentleman SM Striatal Abeta peptide deposition mirrors dementia and differentiates DLB and PDD from other parkinsonian syndromes Neurobiol Dis 2011 41 377 384 10.1016/j.nbd.2010.10.005 20951207

[B65] Gomperts SN Locascio JJ Marquie M Santarlasci AL Rentz DM Maye J Johnson KA Growdon JH Brain amyloid and cognition in Lewy body diseases Mov Disord 2012 27 965 973 10.1002/mds.25048 22693110 PMC3725259

[B66] Villemagne VL Okamura N Pejoska S Drago J Mulligan RS Chetelat G O'Keefe G Jones G Kung HF Pontecorvo M Differential diagnosis in Alzheimer's disease and dementia with Lewy bodies via VMAT2 and amyloid imaging Neurodegener Dis 2012 10 161 165 10.1159/000334535 22261520

[B67] Aarsland D Ballard CG Halliday G Are Parkinson's disease with dementia and dementia with Lewy bodies the same entity? J Geriatr Psychiatry Neurol 2004 17 137 145 10.1177/0891988704267470 15312277

[B68] Woodruff BK Graff-Radford NR Ferman TJ Dickson DW DeLucia MW Crook JE Arvanitakis Z Brassler S Waters C Barker W Duara R Family history of dementia is a risk factor for Lewy body disease Neurology 2006 66 1949 1950 10.1212/01.wnl.0000219812.20616.b3 16801670

[B69] Harding AJ Das A Kril JJ Brooks WS Duffy D Halliday GM Identification of families with cortical Lewy body disease Am J Med Genet B 2004 128B 118 122 10.1002/ajmg.b.30014 15211643

[B70] Nishioka K Wider C Vilarino-Guell C Soto-Ortolaza AI Lincoln SJ Kachergus JM Jasinska-Myga B Ross OA Rajput A Robinson CA Association of alpha-, beta-, and gamma-Synuclein with diffuse lewy body disease Arch Neurol 2010 67 970 975 10.1001/archneurol.2010.177 20697047 PMC4727539

[B71] Ohtake H Limprasert P Fan Y Onodera O Kakita A Takahashi H Bonner LT Tsuang DW Murray IV Lee VM Beta-synuclein gene alterations in dementia with Lewy bodies Neurology 2004 63 805 811 10.1212/01.WNL.0000139870.14385.3C 15365127 PMC1808539

[B72] Zarranz JJ Alegre J Gomez-Esteban JC Lezcano E Ros R Ampuero I Vidal L Hoenicka J Rodriguez O Atares B The new mutation, E46K, of alpha-synuclein causes Parkinson and Lewy body dementia Ann Neurol 2004 55 164 173 10.1002/ana.10795 14755719

[B73] Singleton AB Farrer M Johnson J Singleton A Hague S Kachergus J Hulihan M Peuralinna T Dutra A Nussbaum R alpha-Synuclein locus triplication causes Parkinson's disease Science 2003 302 841 10.1126/science.1090278 14593171

[B74] Higuchi S Arai H Matsushita S Matsui T Kimpara T Takeda A Shirakura K Mutation in the alpha-synuclein gene and sporadic Parkinson's disease, Alzheimer's disease, and dementia with lewy bodies Exp Neurol 1998 153 164 166 10.1006/exnr.1998.6868 9743579

[B75] El-Agnaf OM Curran MD Wallace A Middleton D Murgatroyd C Curtis A Perry R Jaros E Mutation screening in exons 3 and 4 of alpha-synuclein in sporadic Parkinson's and sporadic and familial dementia with Lewy bodies cases Neuroreport 1998 9 3925 3927 10.1097/00001756-199812010-00029 9875730

[B76] Meeus B Verstraeten A Crosiers D Engelborghs S Van den Broeck M Mattheijssens M Peeters K Corsmit E Elinck E Pickut B DLB and PDD: a role for mutations in dementia and Parkinson disease genes? Neurobiol Aging 2012 33 629 e5 629 e18 22118943 10.1016/j.neurobiolaging.2011.10.014

[B77] Mata IF Samii A Schneer SH Roberts JW Griffith A Leis BC Schellenberg GD Sidransky E Bird TD Leverenz JB Glucocerebrosidase gene mutations: a risk factor for Lewy body disorders Arch Neurol 2008 65 379 382 10.1001/archneurol.2007.68 18332251 PMC2826203

[B78] Bogaerts V Engelborghs S Kumar-Singh S Goossens D Pickut B van der Zee J Sleegers K Peeters K Martin JJ Del-Favero J A novel locus for dementia with Lewy bodies: a clinically and genetically heterogeneous disorder Brain 2007 130 2277 2291 10.1093/brain/awm167 17681982

